# Lymphocutaneous Sporotrichosis Refractory to First-Line Treatment

**DOI:** 10.1155/2021/9453701

**Published:** 2021-10-06

**Authors:** Walter Belda, Luiz Felipe Domingues Passero, Ana Thereza Stradioto Casolato

**Affiliations:** ^1^Dermatology Department, University of São Paulo, Medical School, Clinics Hospital, São Paulo, Brazil; ^2^Laboratory of Pathology of Infectious Diseases, Medical School, University of São Paulo, São Paulo, Brazil; ^3^São Paulo State University (UNESP), Institute of Biosciences, São Vicente, Presidente Prudente, Brazil; ^4^São Paulo State University (UNESP), Institute for Advanced Studies of Ocean, São Vicente, Presidente Prudente, Brazil

## Abstract

Sporotrichosis is a fungal infection endemic in Latin America and has been attributed to the thermodimorphic fungus of the genus *Sporothrix*. Transmission to humans occurs during a traumatic injury with soil or organic material; additionally, lesions caused by infected cats play an important role in the epidemiology of the disease. The classic treatment of sporotrichosis is performed with itraconazole or potassium iodide; second-line medications, such as amphotericin B and terbinafine, can alternatively be used in cases of first-line drug failure. In the present study, a patient with lymphocutaneous sporotrichosis in the right upper limb exhibited intolerance to itraconazole and potassium iodide, additionally during the period of use; these drugs did not control skin lesions. In this patient, amphotericin B deoxycholate and its liposomal version were used in this patient; and complete recovery of the lesions was observed.

## 1. Introduction

Sporotrichosis is an infectious disease caused by the thermodimorphic fungus that belongs to the genus *Sporothrix*, and although cosmopolite, it is endemic in Latin America [1]. The main forms of transmission to humans are associated with traumatic injuries that occur with contaminated soil or organic material; infected cats are considered important vectors of this disease and are responsible for epidemic outbreaks in Latin America, such as Brazil [1, 2].

The clinical manifestations of sporotrichosis are classified as cutaneous and extracutaneous. Among the cutaneous forms, sporotrichosis can be further classified as lymphocutaneous, localized cutaneous, and cutaneous caused by multiple inoculations. In extracutaneous clinical form, different organs or systems can be affected. Furthermore, disseminated sporotrichosis can affect the skin, lungs, sinuses, liver, kidney, eyes, heart, and genitalia [3]. Classical sporotrichosis manifests between 2 and 4 weeks after injury and starts as a paponodular lesion at the site of inoculation and develops into ulcerated nodules that drain the seropurulent material. Frequently, this lesion spreads, and more nodules can be formed following the lymphatic vessels.

The gold standard for diagnosing sporotrichosis is to identify and isolate the etiologic agent, *Sporothrix* sp., from cutaneous lesions. Histopathological analysis can be suggestive, but it has low sensitivity due to a reduced number of fungal entities; however, it can increase after staining the tissue with PAS or Gomori; immunolabeling the etiologic agent in histological sections can also enhance the sensitivity of diagnosis [2].

Oral itraconazole, potassium iodide, and terbinafine as well as intravenous amphotericin B have been used in the treatment of sporotrichosis. In cutaneous and lymphocutaneous forms itraconazole is recommended as the first-choice drug for all patients [3, 4] at 200 mg per day until clinical cure. Patients receiving itraconazole therapy exhibit some tolerable side effects, such as headaches, nausea, vomiting, and epigastric pain. In cases of failure of treatment, 400 mg of itraconazole is used per day, divided into two doses. Additionally, itraconazole can be replaced by potassium iodide, initially with 3–5 drops three times a day, until 40–50 drops (4–6 g/day). This drug was chosen as the first-choice treatment for lymphocutaneous and localized clinical forms of sporotrichosis, but was replaced by itraconazole because the dose, counted in drops, is not as accurate as itraconazole. In addition, frequent side effects of potassium iodide include metallic taste in the mouth and gastrointestinal intolerance [3, 4]. Fluconazole has been considered the second-line treatment, but the efficacy of this drug is low compared to itraconazole and potassium iodide [5]. In severe forms of sporotrichosis, amphotericin B deoxycholate or the liposomal version has been used as the first-line drug in treatment, but after improvement of the lesions, it is replaced with itraconazole [6]; furthermore, patients with a compromised immune system also may be treated with amphotericin B when presenting with fixed cutaneous sporotrichosis [7]. In patients, amphotericin B can induce mild and severe side effects, such as fever, chills, headache, malaise, hypokalemia, hypomagnesemia, cardiotoxicity, and nephrotoxicity [8], which in fact is a huge drawback associated with the use of such a drug.

In the present case report, a patient with lymphocutaneous sporotrichosis, intolerant to first-line drugs, potassium iodide and itraconazole, was successfully treated with amphotericin B and presented a significant improvement of lesions.

## 2. Case Report

**Figure 1:**
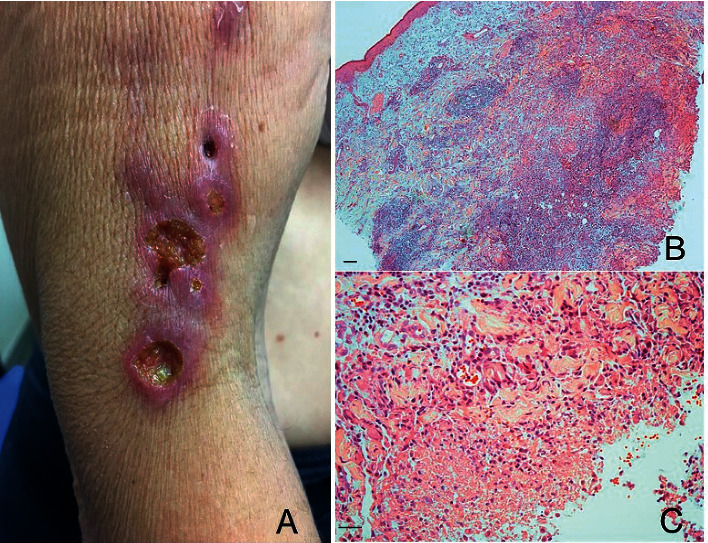
(a) Ulcerated lesions with borders showing erythema and infiltration. (b) Histological section showing the dense inflammatory infiltrate with the presence of granulomas; magnification 100x. (c) Focal necrosis and neutrophil exudation; magnification 200 ×. Histological sections were stained with hematoxylin and eosin. Bars = 5 *μ*m.

The case was a female patient, 61 years old, with lesions in the right upper limb that began 8 months before her appointment in the medical service of Hospital das Clínicas, Faculdade de Medicina da Universidade de São Paulo, Brazil. She was born in Barros Mendes (Bahia, Brazil), but residing in São Paulo city (São Paulo State, Brazil) for the last 45 years, working as a teacher. She has three healthy cats that regularly visit vets; according to her report, the cats did not scratch her before the onset of cutaneous lesions. One year before the appearance of skin lesions, she traveled to the countryside of São Paulo state (Registro town), and although she had contact with the Atlantic forest, skin injury was not observed. Additionally, between travel and appointment at the Hospital das Clínicas, she had no skin injuries or contact with plants and soil. One month after the appearance of the lesion, she was admitted to the Dermatology Service of the Hospital das Clinicas. The lesions were ulcerated, and the borders showed erythema and were infiltrated; the lesions were also covered with yellowish and hematic crusts. Furthermore, some ascendant subcutaneous nodular lesions were detected that suggest lymphangitis in the right upper limb (Figure 1(a)). Mobile and painless lymphadenopathy was detected in the right axillary area. The patient complained of pain in the lesions, and discrete hyaline exudation was verified, and no other local and systemic symptoms were observed. Skin fragments were collected to identify the etiologic agent of the lesions, as the morphology of the lesions suggested lymphocutaneous sporotrichosis, skin tuberculosis, or leishmaniasis. The histopathological study showed chronic granulomatous dermatitis with focal necrosis and neutrophil exudation of neutrophils (Figures 1(b) and 1(c)).

**Figure 2:**
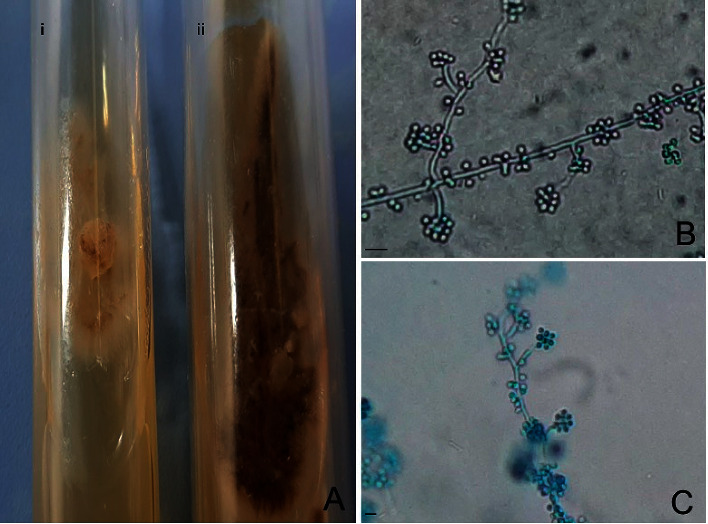
Skin samples were cultured in an agar-dextrose Sabouraud medium. (a) (i) In the sample cultured at 37°C, the fungi presented whitish/yellowish color with leveduriform aspect, and (ii) at 25°C, the fungi presented a brownish color that corresponded to the filamentous form of (b) fungi with conidiophore bouquet-like structures (c), magnification of 400x and 200x, respectively. Bars = 5 *μ*m.

Fite-Faraco and Grocott staining did not evidence the presence of alcohol-acid-resistant bacilli or fungi, respectively. The immunohistochemistry reaction was not positive for BCG. Bacterioscopy and direct fungi research as well as culture of aerobic and anaerobic bacteria were negative. Skin samples cultured in agar-dextrose Sabouraud medium supplemented with chloramphenicol at 25°C allowed the growth of a whitish membranous culture, with a blackened peripheral halo (Figure 2(a)). Microscopically septate hyaline hyphae present in the apex conidiophores and bouquet-like structures were observed, suggesting a fungus belonging to the genus *Sporothrix* (Figures 2(b) and 2(c)). At 37°C, a white/yellowish colony with a smooth aspect was observed; additionally, yeasts were visualized at this temperature. Techniques of molecular biology were not used to identify species.

**Figure 3:**
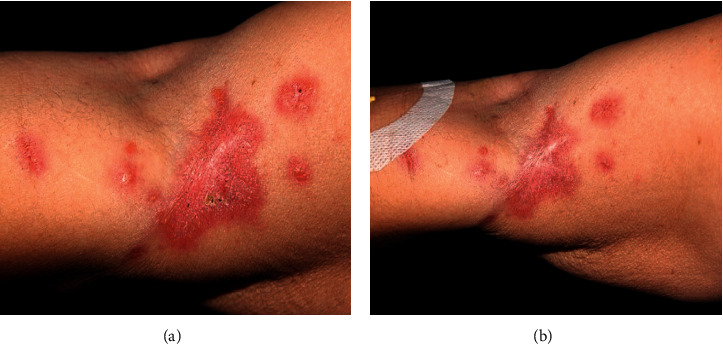
(a) Morphological aspect of the lesions after infusion of 150 mg of amphotericin B deoxycholate and 2600 mg of liposomal amphotericin B presenting re-epithelization and absence of exudate. (b) Morphology of the lesion after infusion of 2600 mg of liposomal amphotericin B and 150 mg of amphotericin B deoxycholate, showing complete re-epithelization of ulcerated lesions and absence of exudation.

**Figure 4:**
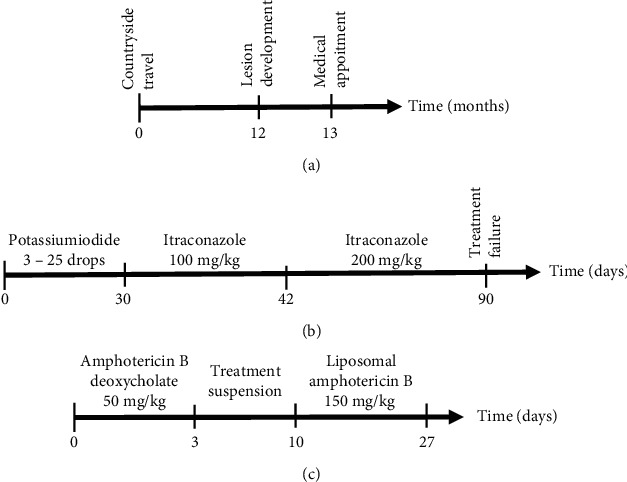
Timeline describing the appearance of the lesion (a); treatment with the first-line drugs potassium iodide and intraconazole (b); and treatment with amphotericin B (c).

Based on these findings, lymphocutaneous sporotrichosis was diagnosed, and potassium iodide was prescribed at 1 g/mL; it was started with three drops per day and gradually increased as the patient tolerated the metallic taste. After one month of treatment, and using 25 drops of potassium iodide per day, the patient reported lack of appetite, nausea, and vomiting. Potassium iodide was replaced by itraconazole, which was administered orally for 12 days at 100 mg/day; as the patient became tolerant, the dose of this antifungal drug was increased to 200 mg/day. The patient reported constant nausea and emetic events after seven days of treatment; however, levels of alanine and aspartate transaminases, alkaline phosphatase, and gamma-glutamyl transferase were normal. Fifty days after the beginning of itraconazole therapy, the patient reported severe gastrointestinal alterations, and the intravenous antiemetic drug metoclopramide was administered to the patient; constant hydration was recommended. Ten days after this event, no improvement of skin lesions was observed. Due to the side effects of both first-line drugs and the resistance of the patient to take oral medicines, such as terbinafine or fluconazole, she was admitted to the Hospital das Clínicas to initiate a treatment with systemic amphotericin B. Additionally, it is important to observe that all these side effects were associated with the therapy with potassium iodide or itraconazole since the patient had no underlying diseases. Electrolytes, renal, and hepatic biochemical tests as well as electrocardiogram were performed, before beginning treatment. Amphotericin B deoxycholate at 50 mg/day (diluted in 500 mL of sodium chloride 0.9% and glucose 5% solution for 6 hours) was given during three days due to the inaccessibility of the liposomal formulation. At the beginning of treatment, episodes of nausea and vomiting were reported, but antiemetic medicines controlled these side effects. Biochemical tests were performed and revealed an increase of serum potassium and creatinine. Correction of electrolyte disturbance was carried out; and to compensate the biochemical alterations, the treatment was suspended for three days. A second set of treatment was performed with liposomal amphotericin B seven days after the infusion with amphotericin B deoxycholate, and it was administered at 150 mg/day (diluted in 500 mL of sodium chloride 0.9% and glucose 5% solution for 6 hours) during 17 days. Clinical improvement of the lesions was observed during treatment (Figures 3(a) and 3(b)). A timeline containing the lesion appearance, diagnosis (Figure 4(a)), and treatment with iodide potassium, itraconazole (Figure 4(b)), or amphotericin B (Figure 4(c)) summarizes the whole treatment performed in this patient. The treatment with liposomal amphotericin B did not change the levels of blood biochemical parameters.

The patient was infused with a total dose of 2600 mg of liposomal amphotericin B and 150 mg of amphotericin B deoxycholate until complete clinical cure, with re-epithelization of the ulcerated lesion and involution of lymphadenopathy. The follow-up has been performed monthly, and relapses were not recorded.

## 3. Discussion

Itraconazole has been used as first-line treatment in cutaneous and lymphocutaneous forms of sporotrichosis [9]. Similarly, potassium iodide, although an old drug, has been efficient at treating human cases [10]. Side effects of such drugs have been associated with gastrointestinal toxicity, as reported by the patient who was intolerant and presented lack of appetite, nausea, and vomiting without alteration of biochemical parameters [11]. Although highly toxic, amphotericin B is prescribed for patients with severe clinical forms, such as disseminated sporotrichosis [12–14]. Despite the toxicity of amphotericin B, this drug was used in lymphocutaneous clinical forms exclusively because the patient was intolerant to both first-line drug options. Although not the standard for the lymphocutaneous clinical form, intravenous treatment with amphotericin B was maintained, while improvement in clinical signals was observed. Amphotericin B topically applied was effective at healing fixed cutaneous sporotrichosis in an immunosuppressed patient that did not tolerate the first-line treatment [7]. In the present report, although the patient has no underlying diseases, the only tolerated treatment was amphotericin B. However, further studies need to be performed to understand the reasons of the refractoriness of the patient to the first-line drugs. Based on these findings, amphotericin B can be considered an interesting example of a successful nonconventional way to treat lymphocutaneous sporotrichosis in patients intolerant to first-line drugs.

## Data Availability

The data used to support the findings of this study are available from the corresponding author upon request.

## References

[1] Orofino-Costa R., de Macedo P. M., Rodrigues A. M., Bernardes-Engemann A. R. (2017). Sporotrichosis: an update on epidemiology, etiopathogenesis, laboratory and clinical therapeutics. *Anais Brasileiros de Dermatologia*.

[2] Arenas R., Sánchez-Cardenas C., Ramirez-Hobak L., Ruíz Arriaga L., Vega Memije M. (2018). Sporotrichosis: from KOH to molecular biology. *Journal of Fungi*.

[3] Mahajan V. K. (2014). Sporotrichosis: an overview and therapeutic options. *Dermatology Research and Practice*.

[4] Costa R. O., de Macedo P. M., Carvalhal A., Bernardes-Engemann A. R. (2013). Use of potassium iodide in dermatology: updates on an old drug. *Anais Brasileiros de Dermatologia*.

[5] García Carnero L., Lozoya Pérez N., González Hernández S., Martínez Álvarez J. (2018). Immunity and treatment of sporotrichosis. *Journal of Fungi*.

[6] Verma G., Verma S., Rattan R. (2019). Lymphocutaneous sporotrichosis of face with verrucous lesions: a case report. *Indian Dermatology Online Journal*.

[7] Mahajan V. K., Mehta K. S., Chauhan P. S., Gupta M., Sharma R., Rawat R. (2015). Fixed cutaneous sporotrichosis treated with topical amphotericin B in an immune suppressed patient. *Medical Mycology Case Reports*.

[8] Laniado-Laborín R., Cabrales-Vargas M. N. (2009). Amphotericin B: side effects and toxicity. *Revista Iberoamericana de Micología*.

[9] de Lima Barros M. B., de Almeida Paes R., Schubach A. O. (2011). *Sporothrix schenckii* and sporotrichosis. *Clinical Microbiology Reviews*.

[10] Xue S., Gu R., Wu T., Zhang M., Wang X. (2009). *Oral potassium iodide for the treatment of sporotrichosis*.

[11] Xue S. L., Li L. (2009). Oral potassium iodide for the treatment of sporotrichosis. *Mycopathologia*.

[12] Kauffman C. A., Bustamante B., Chapman S. W., Pappas P. G. (2007). Clinical practice guidelines for the management of sporotrichosis: 2007 update by the infectious diseases society of America. *Clinical Infectious Diseases*.

[13] Bunce P. E., Yang L., Chun S., Zhang S. X., Trinkaus M. A., Matukas L. M. (2012). Disseminated sporotrichosis in a patient with hairy cell leukemia treated with amphotericin B and posaconazole. *Medical Mycology*.

[14] Ishida K., Castro R. A., Torrado J. J. (2018). Efficacy of a poly-aggregated formulation of amphotericin B in treating systemic sporotrichosis caused by *Sporothrix* brasiliensis. *Medical Mycology*.

